# Low-flow oxygen delivery using a nasal cannula for apnoeic oxygenation in children undergoing elective surgery under general anaesthesia: A randomised controlled trial

**DOI:** 10.1097/EA9.0000000000000034

**Published:** 2023-09-21

**Authors:** Sohini Ray, Anju R. Bhalotra, Mona Arya, Kavita R. Sharma

**Affiliations:** From the Department of Anaesthesiology & Intensive Care, Maulana Azad Medical College and Associated Hospitals (SR, ARB, MA) and Department of Anaesthesiology, Vardhaman Mahavir Medical College and Safdarjung Hospital, New Delhi, India (KRS)

## Abstract

**BACKGROUND:**

Children have a smaller functional residual capacity and higher rate of oxygen consumption making them prone to develop hypoxaemia during a period of apnoea. The effectiveness of apnoeic oxygenation in preventing oxygen desaturation during laryngoscopy and intubation in small children has not been well studied.

**OBJECTIVE:**

To assess the effectiveness of apnoeic oxygenation using a nasal cannula in preventing oxygen desaturation during laryngoscopy and intubation in children.

**DESIGN:**

Prospective randomised double-blind controlled trial.

**SETTING:**

Tertiary care teaching hospital between January 2020 and September 2021.

**PATIENTS:**

One hundred and twenty children of 1 to 6 years age undergoing elective surgery requiring general anaesthesia with endotracheal intubation.

**INTERVENTION:**

Patients were randomly allocated to receive 3 l min^−1^ oxygen by nasal cannula (apnoeic oxygenation group) or no oxygen (control group). Laryngoscopy and intubation was undertaken by a trainee anaesthesiologist.

**MAIN OUTCOME MEASURES:**

The primary outcome was the lowest value of peripheral oxygen saturation (SpO_2_) during laryngoscopy and intubation. Secondary outcomes were the number of children whose SpO_2_ dropped to 95%, from 92% to < 95%, and below 92% and the incidence of bradycardia.

**RESULTS:**

The mean ± SD of lowest SpO_2_ values was 99.95 ± 0.29% in the apnoeic oxygenation group and 98.37 ± 4.60% in the control group (*P* *=* 0.009). No fall of SpO_2_ occurred in any patient in the apnoeic oxygenation group in spite of a longer apnoea time (*P* = 0.012). In the control group, 43 patients had no fall in SpO_2_ (*P* < 0.001), in 12 patients there was a fall in SpO_2_ to 95% (*P* *=* 0.004), in one patient SpO_2_ fell to 92 to <95% (*P* *=* 0.315) and in four patients SpO_2_ fell below 92% (*P* *=* 0.038). There was no incidence of bradycardia.

**CONCLUSION:**

Apnoeic oxygenation using a nasal cannula was effective in preventing oxygen desaturation during laryngoscopy and intubation in children as compared with those who did not receive apnoeic oxygenation.

**TRIAL REGISTRATION:**

Clinical Trials Registry of India: CTRI/2020/01/022724 (www.ctri.nic.in).

**KEY POINTS**

- The mean lowest value of SpO_2_ during laryngoscopy and intubation was significantly higher in children receiving apnoeic oxygenation.
- Use of apnoeic oxygenation resulted in significantly higher values of SpO_2_ during airway management in children.
- The maintenance of high SpO_2_ levels may be especially beneficial during paediatric intubations by anaesthesiology trainees, who may require prolonged or multiple attempts at intubation.

## Introduction

Anaesthetic agents and muscle relaxants used in general anaesthesia inhibit spontaneous respiration, leading to apnoea. During this period of apnoea, the patient is at risk of hypoxaemia, which can have dangerous consequences. For several years, preoxygenation has been the gold standard technique to delay the onset of hypoxaemia and thereby allow more time for airway management.^1,2^ Following the onset of apnoea, hypoxaemia develops more rapidly in children than in adults. This is because of a smaller functional residual capacity, increased closing capacity and a higher rate of oxygen consumption in children.^3–5^ Furthermore, it is often difficult to obtain the co-operation of paediatric patients and achieve effective preoxygenation.

Apnoeic oxygenation is the provision of a continuous oxygen flow during the apnoeic period of airway management, which provides additional time for maintaining safe oxygen saturation.^6,7^ In paediatric intubations, apnoeic oxygenation may help to increase the safe apnoea time.^8,9^ Prolonging the safe apnoea time during paediatric airway management enables safe airway interventions and may reduce the number of intubation attempts, airway injuries, likelihood of hypoxaemia and haemodynamic compromise.^10–12^ An extended safe apnoea time will also reduce the stress and anxiety associated with paediatric airway management, especially in anaesthesiology trainees.^13^

There is a paucity of data on the effectiveness of apnoeic oxygenation in the paediatric age group. We conducted this study to investigate the effectiveness of apnoeic oxygenation using a nasal cannula during laryngoscopy and intubation in healthy children without an anticipated difficult airway who were scheduled for elective surgical procedures under general anaesthesia and in whom the intubation was being undertaken by a trainee anaesthesiologist.

## Materials and methods

### Trial design

This was a randomised parallel-group double-blind controlled trial. Ethical approval for this study was provided by the Institutional Ethics Committee, Maulana Azad Medical College and Associated Hospitals, New Delhi, India (Chairperson Dr A.K. Aggarwal) on 1 November 2019 (approval number F.1/IEC/MAMC/70/05/2019/No 413). The trial was prospectively registered under the Clinical Trials Registry of India (CTRI/2020/01/022724) on 13 January 2020. The trial adheres to the principles of the Declaration of Helsinki. Written and informed consent was obtained from the parents/legal guardians of all patients participating in the trial. This manuscript adheres to the Consolidated Standards of Reporting Trials (CONSORT) guidelines for randomised controlled trials.

### Participants

Children of ages 1 to 6 years of American Society of Anaesthesiologists physical status grade I undergoing elective general, urological, orthopaedic, plastic and ophthalmological surgical procedures under general anaesthesia with endotracheal intubation were included. The study was conducted in the Department of Anaesthesiology and Intensive Care at Maulana Azad Medical College and associated Hospitals, New Delhi, India between January 2020 and September 2021. We excluded patients having active or recent upper respiratory tract infection, cardiac or respiratory disease, obesity, obstructive sleep apnoea, nasal obstruction, tonsillar or adenoid hypertrophy, anticipated difficult airway or requiring nasotracheal intubation.

### Intervention

Patients were randomly allocated 1 : 1 to the two study groups: Group AO (Apnoeic Oxygenation Group) received apnoeic oxygenation (via a nasal cannula at 3 l min^−1^ during laryngoscopy and intubation) and Group C (Control Group) received standard care (no apnoeic oxygenation during laryngoscopy and intubation).

### Primary and secondary outcomes

The primary outcome was the difference in the mean lowest peripheral oxygen saturation (SpO_2_) values between the two study groups. The secondary outcomes were the number of children whose SpO_2_ dropped to 95%, from 92% to < 95% and below 92%, and the incidence of bradycardia.

### Sample size

Based on the study by Olayan *et al.*^8^ assuming a pooled standard deviation of 5.5 units, the present study required a sample size of 53 for each group (total sample size of 106, assuming equal group sizes), to achieve a power of 80% and a level of significance of 5% (two-sided), for detecting a true difference in means between the test and the reference group. We included 120 patients accounting for about 10% attrition.

### Randomisation

Sequence generation was performed by a computer-generated random number table. Allocation into groups was done by opening a sealed opaque envelope immediately before surgery. Patients were asked to pick this envelope from an opaque cardboard box on the day of the surgery by the investigator.

### Blinding

A nasal cannula was placed on the nostrils of patients in both study groups for blinding. The oxygen flow was commenced in patients belonging to group AO by an investigator who was aware of the group allocation while the investigator performing the laryngoscopy and the investigator making observations were unaware of the group allocation.

### Anaesthesia technique

A standard anaesthesia technique was used for all patients. Patients were kept fasting as per ASA guidelines. As per institutional protocol, all patients received premedication with oral midazolam 0.5 mg kg^−1^, 30 min before the surgery. In the operating theatre, standard monitors including pulse oximeter, electrocardiography and noninvasive blood pressure were attached. Baseline values of peripheral oxygen saturation (SpO_2_) and heart rate were noted. General anaesthesia was induced by either the intravenous route (propofol 2 mg kg^−1^ i.v.) or the inhalational route (6 l of oxygen and sevoflurane up to 8%) as thought appropriate for that particular patient. After induction of anaesthesia, an appropriately sized soft nasal cannula was placed in all patients. In group AO, oxygen was given at 3 l min^−1^ via the nasal cannula in all patients. Patients in group C did not receive any oxygen through the nasal cannula, which was placed for the purpose of blinding.

All patients received vecuronium 0.1 mg kg^−1^ i.v. for neuromuscular blockade. Bag and mask ventilation was done with 100% oxygen and sevoflurane for 3 min using a paediatric circle-absorber breathing circuit with a fresh gas flow of 4 l min^−1^. Laryngoscopy and intubation was then performed using an appropriately sized Macintosh laryngoscope by a trainee anaesthesiologist resident under the supervision of a senior consultant anaesthesiologist. Successful intubation was confirmed by auscultation and the appearance of a square wave capnograph. Subsequent anaesthesia care after successful endotracheal intubation was then left to the discretion of the conducting anaesthesiologist. Trainee anaesthesiologists included anaesthesia residents with 12 to 18 months experience in anaesthesia training.

If, during the attempt of intubation, the SpO_2_ fell below 92%, bag mask ventilation with 100% oxygen was to be commenced, and the procedure was to be taken over by the supervising consultant anaesthesiologist. The apnoea time or time to first event was defined as the time taken to either secure the airway as signified by the appearance of a square wave capnogram or the time for SpO_2_ values to fall below 92% at which time bag mask ventilation was to be commenced.

Bradycardia was defined as a heart rate less than 80 bpm in children 3 years or less of age and less than 70 bpm in children older than 3 years age. Any incidence of bradycardia was to be treated with atropine 20 μg kg^−1^. In case for some reason, endotracheal intubation was very difficult or not feasible, the case was to be excluded and managed as per standard guidelines for management of the unanticipated difficult airway in children.

### Statistical methods

Statistical analysis was performed using SPSS Statistical Software version 25.0. Clinical parameters were presented in terms of mean ± SD for quantitative variables and frequency (%) for qualitative variables. Student's *t* test was used to compare the mean difference of continuous parameters between the intervention and control groups. Statistical significance was taken as *P* less than 0.05.

## Results

From 15 January 2020 to 1 September 2021,132 patients were assessed for eligibility for this study. Eleven patients refused to participate and 1 patient did not meet the inclusion criteria. One hundred and twenty patients were enrolled for the trial and were randomly divided between group AO and group C, each group having 60 patients. No patients were lost to follow-up.

The two groups were comparable with respect to characteristics such as age, sex, weight and height (Table 1). The mean baseline SpO_2_ was 99.75 ± 0.57% in group C and 99.53 ± 0.95% in group AO (*P* *=* 0.132). The mean SpO_2_ value after bag mask ventilation and at mask removal was 99.98 ± 0.13% in group C and 99.95 ± 0.29% in group AO, which was also similar in both groups (*P* *=* 0.413) (Table 1).

**Table 1:** Patient characteristics and SpO_2_ before the apnoea period

Parameter	Group C (*n* = 60)	Group AO (*n* = 60)	*P* value
Age (years)	4.02 ± 1.84	4.33 ± 1.89	0.35
Sex (male : female)	45 : 15	46 : 14	0.83
Weight (kg)	16.78 ± 6.47	15.99 ± 5.01	0.46
Height (cm)	102.30 ± 16.51	102.22 ± 16.82	0.98
baseline SpO_2_ (%)	99.75 ± 0.57%	99.53 ± 0.95%	0.13
SpO_2_ value after bag mask ventilation (%)	99.98 ± 0.13%	99.95 ± 0.29%	0.41

Data are mean ± SD. SpO_2_, peripheral arterial oxygen saturation.

In both groups, there was a significant rise in SpO_2_ values from baseline during bag and mask ventilation but this rise was significantly higher in group AO as compared with group C (Table 2). The presence of the nasal cannula did not create any problems in achieving a good mask seal in any patient.

**Table 2:** Increase in SpO_2_ values with bag mask ventilation

	Baseline SpO_2_ (%)	SpO_2_ (%) after 3 mins BMV	*P* value
Group C	99.75 ± 0.57	99.98 ± 0.13	0.002
Group AO	99.53 ± 0.95	99.95 ± 0.29	<0.001

Data are mean ± SD. AO, apnoeic oxygenation; BMV, bag mask ventilation; C, control; SpO_2_, peripheral arterial oxygen saturation.

### Primary outcome

The mean lowest value of SpO_2_ during laryngoscopy and intubation was 99.95 ± 0.29% in group AO, which was significantly higher than the mean lowest value of SpO_2_ in group C, which was 98.37 ± 4.60% (*P* *=* 0.009).

### Secondary outcomes

No fall in the SpO_2_ was seen in any of the 60 children in group AO and 43 patients in group C. This difference was highly statistically significant (*P* < 0.001). In group C, 17 children had a fall in SpO_2_ values during laryngoscopy and intubation. Of these, there was a fall in SpO_2_ to 95% in 12 children, a fall in SpO_2_ to 92 to <95% in 1 child and a fall in SpO_2_ to less than 92% in 4 children (Table 3).

**Table 3:** Fall in SpO_2_ values during laryngoscopy and intubation

	Group C	Group AO	*P* value
No fall in SpO_2_	43	60	<0.001
SpO_2_ fall to 95%	12	0	0.004
SpO_2_ fall to 92 to <95%	1	0	0.31 (NS)
SpO_2_ fall below 92%	4	0	0.038

Data are number of patients. AO, apnoeic oxygenation; C, control; SpO_2_, peripheral arterial oxygen saturation.

The apnoea time was found to be significantly shorter in group C, being 43.78 ± 27.92 s compared with 61.58 ± 46.58 s in group AO (*P* = 0.012). During the apnoea period from mask removal (after 3 min of bag mask ventilation) to the appearance of the first square wave capnogram, there were statistically significant falls in SpO_2_ in group C (*P* *=* 0.014) but there was no change in SpO_2_ in group AO (Table 4). All 60 patients in group AO and 56 patients in group C had single attempt intubations in this study. In four patients in group C, the SpO_2_ fell below 92% at which point the procedure was taken over and bag and mask ventilation with 100% oxygen was commenced by the senior consultant anaesthesiologist. These four patients were intubated at the second attempt.

**Table 4:** SpO_2_ values during the apnoea period

	SpO_2_ (%) after 3 min BMV	SpO_2_ (%) at introduction of endotracheal tube	*P* value
Group C	99.98 ± 0.13	98.51 ± 4.5	0.014
Group AO	99.95 ± 0.29^a^	99.95 ± 0.29	–

Data are mean ± SD. AO, apnoeic oxygenation; BMV, bag mask ventilation; C, control; SpO_2_, peripheral arterial oxygen saturation.

a The correlation and *t* cannot be computed because the standard error of the difference is 0.

The baseline heart rate was 106.32 ± 18.67 bpm in group C and 108.13 ± 19.85 bpm in group AO, which was comparable (*P* *=* 0.607). The lowest value of heart rate in the two groups was also comparable, being 95.55 ± 17.55 bpm in group C and 96.17 ± 17.63 bpm in group AO (*P* *=* 0.848). There was no incidence of bradycardia.

## Discussion

We conducted this study to assess the effectiveness of apnoeic oxygenation using a nasal cannula during laryngoscopy and intubation by trainee anaesthesiologists in ASA-1 children scheduled for elective surgery under general anaesthesia. The lowest value of SpO_2_ during laryngoscopy and intubation was 98.37 ± 4.60% in group C and 99.95 ± 0.29% in group AO (*P* *=* 0.009) in spite of a longer apnoea time, suggesting that the use of apnoeic oxygenation allows higher peripheral oxygen saturation levels to be maintained during the apnoeic period of airway management. Although this difference in SpO_2_ may not be clinically significant, higher values of SpO_2_ can significantly reduce stress in anaesthesia trainees, allow unhurried airway management and minimise the chances of errors.

The baseline SpO_2_ values were similar in both groups, 99.75 ± 0.57% in group C and 99.53 ± 0.95% in group AO. The SpO_2_ values just before mask removal were also comparable, 99.98 ± 0.13% in group C and 99.95 ± 0.29% in group AO. In both groups, there was a significant rise in SpO_2_ from baseline during bag and mask ventilation with 100% oxygen. This significant increase in peripheral arterial oxygen levels can be attributed to the effect of bag and mask ventilation. However, the increase in SpO_2_ from baseline to just before mask removal was higher in group AO, thereby suggesting that nasal oxygenation, when combined with bag and mask ventilation, helps achieve even higher oxygen levels.

The SpO_2_ value just before securing the airway was significantly higher in group AO than group C: being 99.95 ± 0.29 vs. 98.52 ± 4.5% respectively. During the apnoea period from mask removal to securing airway, there were statistically significant falls in SpO_2_ in group C. This was in contrast with group AO, in which there was no change in SpO_2_ from face mask removal until the airway was secured. This suggests that the use of apnoeic oxygenation allows the maintenance of high SpO_2_ values throughout the period of apnoea.

All 60 patients in group AO maintained constant SpO_2_ during airway management as opposed to only 43 patients in group C. The maintenance of SpO_2_ values with no drop at all in SpO_2_ may have allowed the operator to perform the laryngoscopy and intubation in an unhurried manner and this may possibly explain the longer apnoea time seen in this group. The longer time cannot be attributed to the physical presence of the nasal cannula as this was placed in both groups. In four patients in group C, the SpO_2_ fell below 92% at which point bag and mask ventilation was commenced with 100% oxygen. In the meantime, however, the SpO_2_ levels continued to fall and reached 91% in two patients and 70 and 85%, respectively, in the other two patients. Three of these patients were 1 year old and the patient whose SpO_2_ fell to 85% was 4 years old. The one patient, whose SpO_2_ fell to 94% was 5 years old. There seemed to be no correlation with age in the children who had a fall in SpO_2_ during the period of airway management.

In group AO, 58 patients maintained SpO_2_ of 100% throughout the airway management period. In one patient in the apnoeic oxygenation group, the baseline SpO_2_ was 96%, which increased to 98% during bag and mask ventilation and remained at this value throughout the apnoeic period. In another patient, the baseline value of SpO_2_ was 99%, and there was no fall or rise of SpO_2_ from this value throughout the period of airway management. This resulted in a mean SpO_2_ of 99.95% (instead of 100%) just before mask removal and just before securing the airway in group AO. There can be two possible reasons for these SpO_2_ values in these two patients. Firstly, patient-specific factors can play a role. However, as we had included only ASA-1 children in this study without any cardiopulmonary disease, this factor seems unlikely. A second factor can be the reliability of the pulse oximeter used for the study. The sensitivity of pulse oximeters can be affected by the position of the probe, long-standing use, physical damage, accumulation of dirt and nail paint.^14^ The pulse oximeter probe was placed on the finger, which is reliable and valid in measuring oxygen saturation and correlates very closely to arterial blood gas results.^15^ We used the Masimo SpO_2_ module for our study, which has an accuracy of ±2% (measured without motion in adult/paediatric mode), ±3% (measured with motion in adult/paediatric mode) or ±3% (measured without motion in neonatal mode) within the measurement range of 70 to 100%. The accuracy is not specified within 0 to 69%. The SpO_2_ values in these two patients may be a consequence of this inherent inaccuracy of ±2% when using Masimo technology.

### Limitations

Our study has some limitations. We studied only ASA-1 children with normal airways. Further studies are needed to evaluate the benefit of apnoeic oxygenation in children with cardiopulmonary disease or anticipated difficult airway who are more prone to develop hypoxaemia as compared with normal healthy children. We did not measure the safe apnoea time as allowing oxygen desaturation in children is unsafe and unethical. We compared SpO_2_ instead of *P*aO_2_ values. As we know, O_2_ saturation of haemoglobin varies with the *P*aO_2_ in a nonlinear fashion and above 90 mmHg of *P*aO_2_, the curve becomes almost flat, and the rise in SpO_2_ is small despite large increments in *P*aO_2_.^16^ In the present study, as the SpO_2_ of most of the patients remained in the flat upper part of the oxygen-haemoglobin dissociation curve, *P*aO_2_ would have been a more accurate determinant of oxygenation compared with SpO_2_. However, *P*aO_2_ measurement requires serial arterial blood gas analysis, which is not only difficult in children but is also painful, invasive and has the risk of arterial spasm and subsequent gangrene. As we studied ASA-1 children with no cardiopulmonary disease, we did not measure *P*aO_2_. Although blinding of the operator performing the intubation was attempted, the sound of the flow of oxygen in the children in the AO group may have resulted in unintentional unblinding in some cases. Also, we used the same oxygen flow rate of 3 l min^−1^ in all children without any adjustments for age or weight.^17^

### Generalisability

Our study shows the utility of apnoeic oxygenation using a nasal cannula in maintaining high SpO_2_ levels in ASA-1 children. Further studies are needed to assess the utility of apnoeic oxygenation in children with anticipated difficult airway and comorbidities.

## Conclusion

Apnoeic oxygenation resulted in statistically significant higher levels of SpO_2_ at all times during airway management in children. The lowest value of SpO_2_ during laryngoscopy and intubation was significantly higher in the children who received apnoeic oxygenation. Thus, apnoeic oxygenation using a nasal cannula was effective in preventing oxygen desaturation during laryngoscopy and intubation in ASA-1 children.
